# Determinants of female sexual function in inflammatory bowel disease: a survey based cross-sectional analysis

**DOI:** 10.1186/1471-230X-8-45

**Published:** 2008-10-03

**Authors:** Antje Timmer, Daniela Kemptner, Alexandra Bauer, Angela Takses, Claudia Ott, Alois Fürst

**Affiliations:** 1 Department of Medical Biometry and Statistics, University Hospital of Freiburg, Stefan-Meier Strasse 26, 79104, Freiburg, Germany; 2 Landratsamt, Regensburg, Altmühlstraße 3, 93059 Regensburg, Germany; 3 Department of Medicine 1, University Hospital of Regensburg, Franz-Josef-Strauss-Allee 11, 93042 Regensburg, Germany; 4 Department of Surgery, Caritas-Hospital St, Josef Landshuter Straße 65, 93053 Regensburg, Germany

## Abstract

**Background:**

Sexual function is impaired in women with inflammatory bowel disease (IBD) as compared to normal controls. We examined disease specific determinants of different aspects of low sexual function.

**Methods:**

Women with IBD aged 18 to 65 presenting to the university departments of internal medicine and surgery were included. In addition, a random sample from the national patients organization was used (separate analyses). Sexual function was assessed by the Brief Index of Sexual Function in Women, comprising seven different domains of sexuality. Function was considered impaired if subscores were < -1 on a z-normalized scale. Results are presented as age adjusted odds ratios with 95% CI based on multiple logistic regression.

**Results:**

336 questionnaires were included (219 Crohn's disease, 117 ulcerative colitis). Most women reported low sexual activity (63%; 17% none at all, 20% moderate or high activity). Partnership satisfaction was high in spite of low sexual interest in this group. Depressed mood was the strongest predictor of low sexual function scores in all domains. Urban residency and higher socioecomic status had a protective effect. Disease activity was moderately associated with low desire (OR 1.8, 95% CI 1.0 to 3.2). Severity of the disease course impacted most on intercourse frequency (OR 2.3, 95% CI 1.4 to 4.7). Lubrication problems were more common in smokers (OR 2.5, 95% CI 1.3 to 5.1).

**Conclusion:**

Mood disturbances and social environment impacted more on sexual function in women with IBD than disease specific factors. Smoking is associated with lubrication problems.

## Background

Ulcerative colitis and Crohn's disease typically affect adolescents or young adults and are characterized by a chronically remitting course. Many symptoms, complications and consequences of these diseases are likely to impact on body image, intimacy and sexual function [1]. Fatigue, pain and diarrhoe are typical features of relapse and may further be aggravated by the embarrassing features of incontinence and bad odours. Perianal disease affects about one third of patients with Crohn's disease. Many will require surgery, possibly including the installation of a permanent or, more often, transient ostomy. Malnutrition or medical therapy with corticosteroids may also be associated with changes in the bodily appearance. Furthermore, mood disorders, in particular depression, are reported to be common in IBD [2]. Mood disorders are, at the same time, known to constitute a major risk factor for decreased sexual function [3].

It was therefore not surprising, when, almost 15 years ago, Moody and Mayberry described significantly decreased sexual activity in women with Crohn's disease, based on structured interviews in 50 women with friend controls [4]. Twenty-four percent of the cases abstained from sexual activities altogether due to the disease (controls: 4%). Moody and Mayberry complemented this study by another survey including 50 women with ulcerative colitis [5]. Since then, there has been very little research on this issue as recently reviewed [6,7]. The few studies available mostly focused on postoperative outcomes, as abdominal surgery has the potential for structural changes or pelvic nerve damage [8-10]. Many other issues concerning sexual health in women with IBD remain unclear.

We have recently conducted a case control survey in members of the German IBD patients organization [11]. These persons form a relatively healthy group of IBD patients. Even so, all aspects of female sexuality covered by a sensitive validated instrument [12] were impaired in the IBD cases, as compared to controls. In contrast to the results in men, impaired function in women did not depend on the activity of the disease but was also manifest in remission. Due to the case-control design and the use of ambulatory patients only, this previous survey offered little information on disease specific risk factors.

To shed more light on the disease related determinants of sexual function in women with IBD, we have now conducted a survey in a well described clinical sample of patients. Here, we present data from both surveys.

## Methods

### Participants

Consecutive patients with IBD, aged 18 to 65, were included on presentation to the university hospital departments of internal medicine and surgery. Patients with major acute or chronic co-morbidity, unrelated to IBD, were excluded if interference with quality of life and sexuality in particular was considered likely. This was left to the discretion of the treating physician. The list of examples for exclusion included the following: chronic – advanced cancer, complicated diabetes, neurological disease compromising mobility (such as stroke or multiple sclerosis), remitting cardiac failure; acute – recent surgery for any condition other than IBD, all patients currently in or recently transferred from intensive care. Outpatients were also recruited. The response rate was calculated as the proportion of contacted persons returning a completed questionnaire.

This clinical group was complemented by cases from the national patient organization (German Crohn's and Colitis Association/Deutsche Crohn und Colitis Vereinigung, DCCV e.V.). These had been selected randomly from the members' list stratified by type of disease [11].

### Questionnaire

Questionnaires were identical for the two groups (clinical group and national patient organization) with respect to the assessment of exposures and sexual function. For clinical patients additional modules on disease specific quality of life were included (Short Inflammatory Bowel Disease Questionnaire (S-IBDQ) [13], QLQ CR 38 module of the European Organization for Research and Treatment of Cancer (EORTC) [14].

Information on sociodemographic data, life style factors, co-morbidity, and co-medication was collected using a questionnaire module developed by the German IBD Competence Network for use in IBD patients. Life style factors included sports, smoking and alcohol consumption. Socioeconomic status (SES) was calculated as a composite measure based on income, education and professional status, as suggested by the German Epidemiological Association, DGEpi [15], and were summarized as low, middle, and high.

Specific questions related to the presence of diabetes and hypertension. Other co-morbidities and co-medications were evaluated based on open questions. Diseases (such as cardiovascular, hepatobiliary) and medications (such as antihypertensive, antidepressant, other psychiatric) were coded, grouped and used for a summary co-morbidity measure: minor: one disease OR one medication; major: a) at least one disease AND at least one medication or b) more than one disease or medication.

The disease activity was classified as remission/low activity vs. moderate/high activity. The cut points were set at </> 220 for Crohn's disease on a modified Crohn's Disease Activity Index for use in surveys (S-CDAI), and </> 5 for ulcerative colitis on the Clinical Activity Index survey version (S-CAI). This instrument was previously validated [16]. Disease course (severity) was graded as mild (less than one relapse per year), medium (1 or 2 relapses per year) and severe (frequent relapses or persistent problems). Psychological functioning was assessed with the Hospital Anxiety and Depression Scale (HADS) [17,18]. Persons were considered depressed or anxious if the respective HADS-Subscore exceeded 10.

### Assessment of sexual function

As there was no validated questionnaire on female sexual dysfunction available for the use in German language, the Brief Index of Sexual Function in Women (BISF-W) was transformed for use in German [12,19]. Formal cultural adaptation methods were used as described for other quality of life instruments [20]. This included independent translation of the questionnaire by two native speakers of German and back-translation into English by a native speaker of English. Further minor modifications of the layout and wording were applied following a pilot study in 60 persons (patients with and without IBD, healthy volunteers) to improve understandibility and ease of use. The questionnaire then underwent formal validation assessments, including test-retest reliability, internal concistency, construct validity and sensitivity for change [21]. Overall, the BISF-W showed excellent test properties for all domains but the subscore of *specific sexual problems*, which lacked internal consistency. This domain combines several, mainly somatic problems occuring with sexual intercourse in women, such as vaginismus, bleeding or headaches after intercourse.

For subscores and the total score, higher values denote better function with the exception of the *specific sexual problems *(higher score = more problems). The scores were calculated and transformed as suggested in the key-publication by Mazer [12]. Due to a layout problem in the questionnaire, one of two items pertaining to the *arousal *domain was incorrectly or not at all answered by the majority of patients. *Arousal *scores will therefore be omitted from the subscore presentation in this paper due to limited interpretability. Tests on correctly completed questionnaires showed that total scores were reliably estimated without the missing item (correlation coefficient: r = 0.99).

Results from a healthy German control group were used as a standard population (friend controls from our matched case control study). Due to a negative correlation with age (in particular, for the subscores of intercourse frequency and thoughts and desires), standardization was stratified by age. Z-transformation results in a score where 0 represents the standard population mean, and 1 the standard deviation. Low sexual function in any of the domains was defined as a score < -1, representing a score lower than one standard deviation from the mean score of the normative sample.

The EORTC module for colorectal cancer patients was chosen as it contains several direct questions on body image, sexual function and satisfaction and ostomy function [14]. It generally applies four answer categories, which were collapsed into two and presented as the proportion of those answering definitely or rather positive (vs. definitely or rather negative) for simplified graphical display. Although previously used in IBD patients [22], the instrument has not been developed and validated for this purpose. Therefore, summary scores will not be presented.

### Statistical analyses

For exploratory analyses graphical displays (means with 95% confidence intervals (95% CI)) are used. In addition, the frequency of low scores is reported separately for both study groups for descriptive purposes. As crude rates are not comparable between women with and without partners these figures are presented for women with partners only. However, all women were included in the main analyses.

Multivariate logistic regression was applied to calculate adjusted odds ratios (OR) with 95% CI (SPSS^©^) [23]. Increased ratios (OR > 1.0) denote an increased risk for low function in participants positive for the respective risk factor. In the multivariate analyses, full models were initially calculated, including all potential risk factors, followed by manual stepwise elimination based on model fit, and the robustness and the statistical significance of the estimated coefficients. All models were adjusted for age and having a partner.

### Ethical considerations

The study was approved by the ethics committee of the institution (02/163). The steering board of the patients' organization also approved of the protocol, questionnaires and information material. Clinical patients provided written consent. Patient organization members consented by returning a completed questionnaire based on written information.

## Results

### Participants

Clinical patient recruitment took place from September 1, 2003 through October 31, 2005. 119 eligible women were approached, 107 agreed to participate (90%). Of these, 95 returned a completed questionnaire (clinical sample response rate 81%). No patient related characteristics were found to be associated with response in this sample.

In addition, nineteen questionnaires from the outpatient department were received. For this group, a response rate could not be calculated as recruitment had to be paused repeatedly due to interference with competing clinical trials (unknown denominator).

Of the patient organization member survey, 222 questionnaires were available for analysis (500 women selected; response rate 44%). In this sample, the response rate was higher in younger women (< 30 years of age: 58%). The combined sample comprised 336 women. 219 of these (65%) had Crohn's disease, 117 ulcerative colitis. The sociodemographic and other characteristics by disease type and patient group are shown in Table 1. Patients in the clinical group were younger. The regional East-Bavarian catchment area of the university hospital is reflected by high proportions of rural residency and catholicism. Clinical patients were also more often depressed and more often smokers. As expected, smoking status was dependent on the type of disease (less smokers in ulcerative colitis).

**Table 1:** Participants' characteristics – demographics and other general information

	Group A – clinical		Group B – DCCV	
	Crohn's disease	Ulcerative colitis	Crohn's disease	Ulcerative colitis

Age (median)	38.5 yrs	38.0 yrs	38.0 yrs	38.0 yrs
Working fulltime	32 (36%)	5 (21%)	40 (31%)	31 (33%)
In training/at school	5 (6%)	1 (4%)	10 (8%)	5 (5%)
SES high	8 (10%)	4 (20%)	48 (39%)	35 (40%)
SES low	31 (38%)	5 (25%)	13 (11%)	3 (3%)
Urban residency	13 (15%)	5 (21%)	51 (40%)	36 (39%)
Smokers	46 (52%)	6 (25%)	38 (30%)	6 (7%)
Roman catholic	74 (82%)	15 (63%)	35 (27%)	27 (29%)
Protestant/Lutheran	9 (10%)	5 (21%)	55 (43%)	33 (36%)
Comorbidity, minor	19 (21%)	4 (3%)	20 (16%)	14 (15%)
Comorbidity, major	22 (24%)	5 (21%)	31 (24%)	24 (26%)
Diabetes	5 (6%)	6 (3%)	1 (1%)	2 (2%)
Antihypertensive Therapy	7 (8%)	4 (17%)	7 (5%)	5 (5%)
On hormonal contraception	25 (28%)	6 (25%)	29 (22%)	24 (26%)
On hormone replacement	3 (3%)	1 (4%)	5 (4%)	4 (3%)
Anxiety	33 (37%)	7 (30%)	38 (30%)	20 (22%)
Depression	19 (21%)	8 (35%)	12 (10%)	9 (10%)
Has a partner	61 (69%)	18 (78%)	93 (74%)	74 (80%)
Married	41 (46%)	16 (67%)	65 (51%)	53 (57%)

N	90	24	129	93

Not all categories shown for most variables.

The comparison of the disease specific characteristics revealed the expected higher proportion of patients with severe or active disease and those on steroids in the clinical group (Table 2). Long term immunosuppression, on the other hand, was more common in the DCCV group, which was also characterized by a longer disease duration and higher percentage of past resecting surgery.

**Table 2:** Participants' characteristics – disease related information (self reported)

	Group A – clinical		Group B – DCCV	
	Crohn's disease	Ulcerative colitis	Crohn's disease	Ulcerative colitis

Disease duration (median)	9.0 yrs	6.0 yrs	13.0 yrs	9.0 yrs
Disease onset > 40 yrs	14 (16%)	3 (13%)	7 (6%)	6 (7%)
Disease onset < 18 yrs	16 (18%)	3 (13%)	28 (22%)	15 (17%)
Active disease	38 (42%)	19 (80%)	28 (22%)	31 (35%)
Mild disease course	22 (26%)	5 (23%)	44 (36%)	32 (37%)
Severe disease course	35 (42%)	7 (32%)	45 (37%)	21 (24%)
Past resecting surgery	38 (42%)	1 (4%)	73 (57%)	10 (11%)
Ostomy	10 (11%)	1 (4%)	13 (10%)	4 (5%)
Ileocecal resection	27 (30%)		48 (37%)	
Perianal disease, current	19 (22%)		26 (21%)	
Perianal disease, ever	23 (27%)		36 (28%)	
Currently on steroids	39 (44%)	11 (48%)	35 (28%)	25 (28%)
Other immunosuppression	18 (20%)	6 (25%)	43 (34%)	19 (21%)
Total/substantial Colitis		14 (64%)		44 (49%)
Left sided colitis		5 (23%)		29 (33%)
Proctitis only		3 (14%)		16 (18%)
Small bowel only	14 (19%)		25 (20%)	
Large bowel only	14 (19%)		37 (29%)	
Combined disease	41 (56%)		61 (48%)	
Upper Gi Involvement	4 (5%)		5 (4%)	

N	90	24	129	93

Not all categories shown for most variables, missing values not reported

### Sexual function – explorative analyses

**Figure 1:**
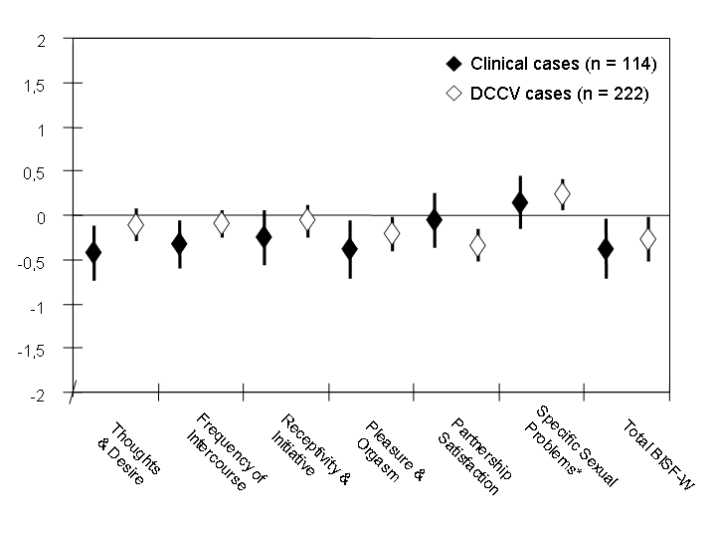
**BISF-W scores (z-values) by case group, women with partners only.** 0: standard population mean, +/- 1 = +/- 1 standard deviation from standard population mean. *specific problems: higher value = more problems, other scores: higher value = better function.

In Figure 1, mean z-values for BISF-W subscores and the total score are shown by study group with 95% CI, restricted to women with partners for better comparability. Case scores were consistently lower (or higher for *specific problems*) than 0 (representing the standard population mean), but all deviations were minor (within one standard deviation). For most subscores, patient organization members scored slightly better than clinical cases with the notable exception of partnership satisfaction. Differences between the groups were not statistically significant for any of the subscores.

**Figure 2:**
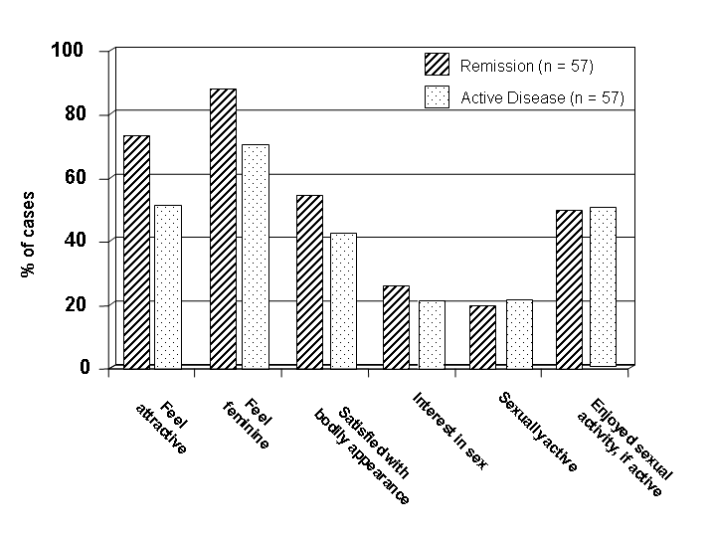
**Results from EORTC-QLQ CR30 items, clinical sample only (n = 114).** Proportions of those answering "very"/"moderate" (vs. "little"/"not at all").

On direct questioning (EORTC-items), issues relating to feeling attractive or feminine as well as satisfaction with the bodily appearance showed an association with disease activity (p < 0.05 for all comparisons) (Figure 2, information available for clinical sample only). There was a strikingly low general interest in sexual activities in both patients with active and quiescent disease. Only 20% reported a high or moderate level of sexual activity during the preceding four weeks. The proportion not sexually active at all was 17%.

### Determinants of impaired sexual function

Adjusted odds ratios from multivariate analyses are presented in Table 3 for selected variables. There was no evidence for differences between patients with Crohn's disease and patients with ulcerative colitis. Depressive mood was the only consistent risk factor, with strong associations for all subscores. Anxiety seemed to be of importance for the domain of *intercourse frequency *only. For other characteristics, associations were sparse.

**Table 3:** Determinants of low sexual function

	Thoughts & Desire	Intercourse frequency	Initiative & receptivity	Pleasure & orgasm	Partnership Satisfaction	BISF-W – Total Score
Clinical vs. DCCV case	1.4 (0.8 to 2.4)	1.3 (0.6 to 2.8)	1.3 (0.7 to 2.2)	1.5 (0.9 to 2.6)	0.6 (0.3 to 1.0)	0.9 (0.5 to 1.5)
Ulcerative colitis vs. Crohn's disease	1.3 (0.7 to 2.1)	1.4 (0.8 to 2.7)	1.3 (0.8 to 2.3)	1.3 (0.8 to 2.2)	1.1 (0.7 to 1.9)	1.3 (0.8 to 2.2)
Ostomy	0.8 (0.3 to 2.1)	1.2 (0.5 to 3.0)	1.5 (0.6 to 3.7)	**0.5 (0.2 to 1.3)**	0.9 (0.4 to 2.0)	0.7 (0.3 to 1.8)
Current perianal disease	0.8 (0.4 to 1.7)	1.6 (0.8 to 3.1)	0.7 (0.3 to 1.6)	0.8 (0.4 to 1.7)	1.7 (0.8 to 3.5)	1.0 (0.5 to 2.0)
Active disease	1.8 (1.0 to 3.2)	1.7 (0.9 to 3.1)	1.5 (0.8 to 2.7)	1.2 (0.7 to 2.1)	0.9 (0.5 to 1.6)	0.9 (0.5 to 1.6)
Frequent relapses	1.2 (0.6 to 2.3)	**2.3 (1.4 to 4.7)**	1.6 (0.8 to 3.1)	1.8 (0.9 to 3.5)	1.2 (0.7 to 2.3)	1.3 (0.7 to 2.4)
Disease duration > 10 yrs	0.7 (0.3 to 1.7)	0.6 (0.3 to 1.5)	0.9 (0.4 to 2.1)	0.6 (0.3 to 1.2)	1.2 (0.5 to 2.6)	0.8 (0.4 to 1.7)
Current steroids	1.0 (0.6 to 1.7)	1.9 (1.1 to 3.3)	1.2 (0.7 to 2.0)	**2.5 (1.5 to 4.4)**	1.2 (0.7 to 1.9)	1.0 (0.6 to 1.7)
Urban residency	0.8 (0.4 to 1.5)	0.8 (0.4 to 1.4)	**0.4 (0.2 to 0.8)**	**0.5 (0.3 to 0.9)**	0.9 (0.5 to 1.6)	0.6 (0.3 to 1.0)
High SES	**0.4 (0.2 to 0.8)**	0.6 (0.2 to 1.3)	0.8 (0.3 to 2.0)	1.5 (0.8 to 2.8)	**2.4 (1.0 to 5.5)**	0.8 (0.3 to 1.7)
Co-morbidity (major)	1.4 (0.7 to 2.6)	1.9 (1.0 to 3.5)	1.1 (0.5 to 2.0)	1.5 (0.8 to 2.8)	1.8 (1.0 to 3.2)	1.3 (0.7 to 2.4)
Depression	**2.9 (1.5 to 5.7)**	**3.5 (1.6 to 7.9)**	**4.4 (2.1 to 9.1)**	**4.8 (2.2 to 10.1)**	**2.7 (1.4 to 5.4)**	**3.4 (1.4 to 6.9)**
Anxiety	1.6 (0.9 to 2.7)	**2.0 (1.1 to 3.6)**	1.4 (0.8 to 3.9)	1.7 (1.0 to 3.0)	1.5 (0.8 to 2.6)	1.4 (0.8 to 2.3)

All odds ratios (95% CI) controlled for age, partnership status and determinants significantly contributing to the respective model; n = 336 or less. Strong associations in bold font (OR > 2.0; OR < 0.5)

The multivariate analysis confirmed the difference in *partnership satisfaction *between clinical and DCCV cases: women in the clinical group were less often dissatisfied, even after adjustment for SES, disease activity, depression, age and partnership status (OR 0.6; 95% CI 0.3 to 1.0). Otherwise, there was no evidence for systematic differences between the two groups. Also, there was not difference between women with Crohn's disease and women with ulcerative colitis.

Disease activity contributed to the prediction of a low *thoughts & desire *score (OR 1.8; 95% CI 1.0 to 3.2). The disease course (severity) was more consistently predictive with OR > 1.0 for all subscores and a strong positive association with *intercourse frequency *(OR 2.3). Current use of steroids was associated with low *pleasure & orgasm *scores. For the presence of an ostomy or current perianal disease, associations were not detected for any of the subscores. The direction of the results for these factors was inconsistent, rendering a relevant effect unlikely. Confidence intervals for effects of these infrequent situations were too wide to provide conclusive evidence.

In contrast, a marked differential effect was found for high SES, which was protective of low *thoughts & desire*, but a risk factor for low *partnership satisfaction*. Urban residency showed consistently low odds ratios with strong negative associations for *initiative & receptivity *and *pleasure & orgasm*. Specific lifestyle factors (smoking, sports, alcohol) did not show conclusive associations with any of the subscores.

### Specific sexual problems

As the subscore of specific sexual problems performed poorly as an aggregate measure in the validation assessment (see methods), analyses for this domain were based on answers to single items. Insufficient lubrication was reported to occur at least occasionally in 30% of the participants who had a sexual relationship. Current smoking was identified as a strong (and only) predictor of this problem (OR 2.5, 95% CI 1.3 to 5.1). Pain during intercourse was also quite common (25%), followed in prevalence by vaginal infections (9%) and vaginismusus (8%). Predictors for any of these complaints were not identified. In particular there was no difference by disease type or activity, use of steroids, co-morbidity or presence of perianal disease. Incontinence of urine during intercourse (n = 5, 2%), headaches following sexual activity (n = 5; 2%) and postcoital bleeding (n = 12; 5%) were considered too infrequent for meaningful further exploration or multivariate analysis.

**Figure 3:**
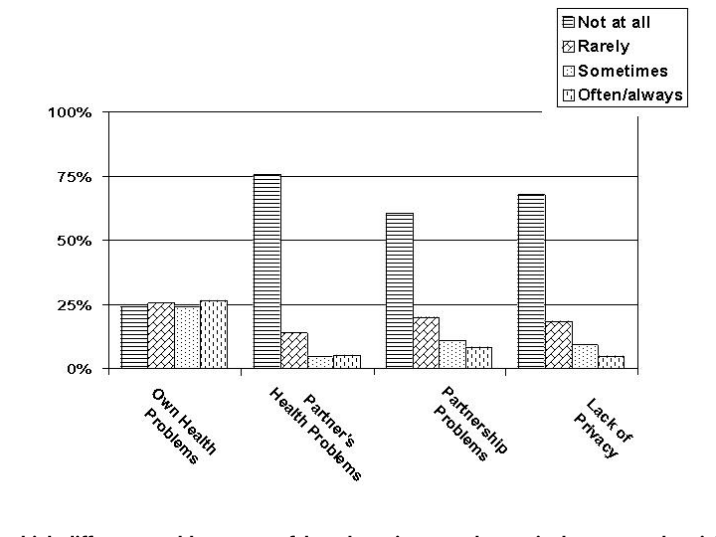
Frequency with which different problems were felt to have impacted negatively on sexual activities (women with partners only).

The extent to which the disease was perceived to have had a negative influence on sexual activities over the last four weeks is displayed relative to other common distractors in Figure 3.

## Discussion

This study was designed to examine disease specific determinants of impaired sexual function in women. The combination of two different case groups enabled us to examine both patients in their normal living environment as well as those patients typically encountered by the university specialist, representing a wide array of clinical problems.

No particular feature of IBD was identified that would explain the predescribed high prevalence of sexual impediments in women suffering from this chronic disease. Rather, the significance of psychosocial factors for female sexuality, well known from studies in normal populations, was further underlined [24,25]. Foremost, depressed mood was confirmed as the strongest and most consistent risk factor. With the exception of *specific sexual problems*, depression impacted on all aspects of sexuality as assessed by the BISF-W. Anxiety seemed less important but may play a role in certain aspects of sexuality (*intercourse frequency*). Urban residency was protective for several problems while the impact of socioeconomic status varied by subscore: Women with a higher socioeconomic status had higher scores for *pleasure & orgasm*, but scored worse on *partnership satisfaction*.

### This subscore was also unique in that it was the only score that differed between the two study groups

The clinical patients were characterized by a higher than average level of *partnership satisfaction*. This phenomenon was underlined by several free-text comments such as "the disease has brought us closer together". Several women expressed gratefulness for their understanding partners who did not let them down in the difficult times of the disease. The importance of an understanding social environment, including the partner, for coping with IBD has been repeatedly observed in studies using analytic interviews, foremost in the context of adjustment to an ostomy [26].

It is unclear why this effect was different in the two groups in spite of control for potential confounders of relationship appreciation. As the DCCV sample suffered from a rather low response rate, responder bias may have caused this observation. Possibly, for some reason, women in unsatisfactory relationships were more likely to respond. Alternatively, it is conceivable that partnership appreciation in stressful situations is regionally different due to cultural factors beyond those assessed (rural residency, confession of faith, SES), or is different in persons joining self help organizations.

The most striking finding in our survey might be the very low levels of sexual interest and activity. A high prevalence of low sexual desire is known from surveys in healthy populations [24,27]. Although low desire is acknowledged as a disease entity in the Diagnostic and Statistical Manual of Mental Disorders (DSM IV – R), it remains controversial whether it constitutes a sexual disorder [28,29]. For example, in women with chronic disease, loss of desire might be considered a physiological response to pain, fatigue or psychological distress. We do not wish to imply that all women with low scores consider themselves deficient or should receive treatment.

It was unfortunate that we were not able to calculate *arousal *subscores for the majority of participants, as some might suspect *arousal *to be particularly sensitive to structural changes or inflammatory processes in the perivaginal and pelvic region. The BISF-W *arousal *domain focuses on the subjective perception of sexual arousal which has been shown to poorly correlate with the physiological responses characterized by genital vasocongestion and lubrication [30]. We were able to assess predictors of lubrication as a somatic aspect of *arousal *and found a strong association with smoking. Confounding by this known risk factor of Crohn's disease should therefore be considered when vaginal dryness in Crohn's disease is reported. We are not aware of any data explaining this effect of smoking on the vaginal mucosa and would welcome any discussion on this issue. There were no disease specific determinants. In particular, there was no epidemiological evidence for a causative effect of immunosuppression, disease activity, or perianal disease in the pathogenesis of this or any of the other more common *specific sexual problems*.

Our findings in women were quite different from our previous observations in men [31]. Erectile function was mainly effected by somatic problems, while mood had more impact on issues of satisfaction. A similarly clear cut differentiation between the somatic and psychosocial aspects of sexuality was not possible in women. This may be innate to the more complex sequence of sexual behavior in women [28]. In addition, our results confirm previously reported observations that women perceive the impact of the disease on sexuality and partnership differently [32].

## Conclusion

In summary, while low sexual function is common in women with IBD, no specific disease related characteristics were identified. Rather, as in women without chronic disease, psychosocial factors play a predominant role. While our data provide some basis for patient counselling, the existence and relevance of sexual problems and any need for therapy should be determined individually in sympathetic patient-doctor interviews, if the patient wishes to do so. Often, treatment of mood disorders might be more important than specific sexual therapy. With respect to the most common specific sexual problem, insufficient lubrication, our study provides yet another reason to advice patients with Crohn's disease against smoking.

## Abbreviations

BISF-W: Brief Index of Sexual Function in Women, CAI: Colitis Activity Index; CDAI: Crohn's Disease Activity Index; EORTC: European Organization for Research and Treatment of Cancer; HADS: Hospital Anxiety and Depression Scale; IBD: Inflammatory bowel disease; QLQ – CR: Quality of Life, Colorectal Cancer; S-CAI: Survey based Colitis Activity Index; S-CDAI: Survey based Crohn's Disease Activity Index; S-IBDQ: Short Inflammatory Bowel Disease Questionnaire.

## Competing interests

The authors declare that they have no competing interests.

## Authors' contributions

AT conceived and planned the study, developed the questionnaire, recruited patients, did the analysis and wrote the manuscript. DK participated in the planning of the study, piloted instruments and recruited patients. AB organized the survey, recruited patients and did some analyses. AT organized clinical recruitment and was responsible for data management. CO participated in patient recruitment. AF participated in the planning of the study, the development of the questionnaire and data retrieval. All authors read and the final version of this manuscript.

## Pre-publication history

The pre-publication history for this paper can be accessed here:


